# Redox-Sensitive Regulation of Myocardin-Related Transcription Factor (MRTF-A) Phosphorylation via Palladin in Vascular Smooth Muscle Cell Differentiation Marker Gene Expression

**DOI:** 10.1371/journal.pone.0153199

**Published:** 2016-04-18

**Authors:** Minyoung Lee, Alejandra San Martín, Alejandra Valdivia, Abel Martin-Garrido, Kathy K. Griendling

**Affiliations:** Department of Medicine, Division of Cardiology, Emory University, Atlanta, Georgia, United Sates of America; Albany Medical College, UNITED STATES

## Abstract

Vascular smooth muscle cells (VSMCs) undergo a phenotypic switch from a differentiated to synthetic phenotype in cardiovascular diseases such as atherosclerosis and restenosis. Our previous studies indicate that transforming growth factor-β (TGF-β) helps to maintain the differentiated phenotype by regulating expression of pro-differentiation genes such as smooth muscle α-actin (SMA) and Calponin (CNN) through reactive oxygen species (ROS) derived from NADPH oxidase 4 (Nox4) in VSMCs. In this study, we investigated the relationship between Nox4 and myocardin-related transcription factor-A (MRTF-A), a transcription factor known to be important in expression of smooth muscle marker genes. Previous work has shown that MRTF-A interacts with the actin-binding protein, palladin, although how this interaction affects MRTF-A function is unclear, as is the role of phosphorylation in MRTF-A activity. We found that Rho kinase (ROCK)-mediated phosphorylation of MRTF-A is a key event in the regulation of SMA and CNN in VSMCs and that this phosphorylation depends upon Nox4-mediated palladin expression. Knockdown of Nox4 using siRNA decreases TGF-β -induced palladin expression and MRTF-A phosphorylation, suggesting redox-sensitive regulation of this signaling pathway. Knockdown of palladin also decreases MRTF-A phosphorylation. These data suggest that Nox4-dependent palladin expression and ROCK regulate phosphorylation of MRTF-A, a critical factor in the regulation of SRF responsive gene expression.

## Introduction

In the vasculature, differentiated vascular smooth muscle cells (VSMCs) are critical for physiological homeostasis; thus, strategies to prevent VSMC de-differentiation are attractive targets for pharmacological intervention. Differentiated VSMCs express SMC-specific contractile proteins including smooth muscle α-actin (SMA) and calponin (CNN) [1]. However, VSMCs undergo the process of dedifferentiation, characterized by decreased differentiation marker gene expression and increased proliferation, migration, and matrix synthesis, in various cardiovascular diseases such as atherosclerosis and in-stent restenosis. Despite decades of research, the molecular mechanisms required for the induction of differentiation marker gene expression in VSMC phenotype remain incompletely understood.

Reactive oxygen species (ROS), such as superoxide and hydrogen peroxide, are implicated in the regulation of signaling pathways involved in VSMC growth, differentiation, migration, and inflammation [2]. While hydrogen peroxide is produced by multiple enzymatic pathways, hydrogen peroxide used in growth- and differentiation-related signaling in aortic VSMCs is derived from NADPH oxidases, Nox1 and Nox4, respectively [2]. TGF- β is a major differentiation factor for smooth muscle [3]. Our previous work has shown that knockdown of Nox4 reduces TGF-β-induced SMA and CNN mRNA and protein expression in VSMCs [4, 5]. Because Nox4 has been found in the nucleus [6], and Nox4 regulates SMA transcription[5], a role for Nox4 in regulation of the transcription factors associated with differentiation marker gene expression is likely.

VSMC contractile gene transcription is largely regulated by serum response factor (SRF), which binds to highly conserved CArG cis-elements (CC(A/T)_6_GG) that are present in the promoter of SMC-specific genes. Transforming growth factor-β (TGF-β), a Nox4 activator, increases differentiation marker gene expression by inducing myocardin or myocardin-related transcription factors (MRTFs)—A and—B binding to SRF[7, 8]. One mechanism of MRTF-A activation encompasses translocation to the nucleus, as has been observed in fibroblasts [9]. In some SMCs, however, the majority of MRTF-A is found in the nucleus [10, 11], suggesting that other signaling mechanisms are needed for the activation of MRTF-A. One such possibility is phosphorylation. MRTF-A has 23 predicted serine/threonine predicted phosphorylation sites (as assessed using Phosphositesite.org), but very little experimental evidence exists regarding the functionality of these sites, their redox-sensitivity, or the upstream kinases that might target MRTF-A. In NIH3T3 cells exposed to serum, MRTF-A phosphorylation is reduced by C3 transferase, a Rho inhibitor, and U0126, an ERK inhibitor [9], while in HeLa cells, MRTF-A is phosphorylated by ERK [12]. However, how phosphorylation contributes to MRTF-A activity in SRF-responsive gene expression remains unclear, especially in VSMCs.

The suggestion that Rho activation is upstream of MRTF-A phosphorylation is intriguing because we have previously shown that Rho is activated by Nox4 [13]. Whether Rho-mediated signaling is responsible for the observed effects of Nox4 on differentiation marker gene expression remains unknown. While the above inhibitor studies suggest that Rho kinase (ROCK) is a potential Nox4 effector, Nox4-mediated regulation of actin dynamics could also play a role [4, 8]. Of the many possible actin-associated proteins that might act downstream of Nox4, palladin is of particular importance because it has been linked to VSMC differentiation during development and *in vitro* [14, 15]. Palladin is an actin-associated protein widely expressed in actin-based subcellular structures [16] including stress fibers, focal adhesions, cell-cell junctions, and embryonic Z-lines [17, 18]. Seven isoforms of palladin have been found in different types of cells, but the 90-kDa and 140-kDa isoforms of palladin are highly expressed in SMCs [19]. Co-immunoprecipitation assays (co-IP) in HEK 293 cells show that palladin binds to MRTFs but not myocardin. Immunocytochemistry assays clearly show that the C-terminal fragment, but not the N-terminal fragment, of palladin co-localizes with MRTF-A in the nucleus [14].

Based on these observations, we hypothesized that in VSMC, redox control of MRTF-A phosphorylation is a major mechanism regulating TGF-β-induced SMA and CNN expression. As we confirmed this hypothesis, we identified the actin binding protein palladin as a downstream effector of Nox4, required for ROCK-mediated phosphorylation of MRTF-A in response to TGF-β.

## Materials and Methods

### Materials

TGF-β was obtained from R&D Systems (Minneapolis, MN, USA). Goat anti-MRTF-A (C-19) and rabbit anti-MRTF-A (H-140) antibodies (Santa Cruz Biotechnology, Santa Cruz, CA, USA); rabbit anti-palladin, mouse anti-SMA, mouse anti-calponin, mouse anti-β-tubulin, and mouse anti-β-actin antibodies (Sigma, St. Louis, MO, USA); and phospho-Ser/Thr antibody (Abcam, Cambridge, MA) were used for western blot. siNox4, siMRTF-A, siPalladin and All-Star negative control siRNA (siNeg) (Qiagen, Valencia, CA), Lipofectamine RNAiMAX (Invitrogen, Mountain View, CA), and OPTI-MEM (Gibco) were used for siRNA transfection. ROCK inhibitor (Y-27632) (Sigma, St. Louis, MO, USA) was used for kinase inhibition.

### Cell Culture

Human aortic smooth muscle cells (here referred to as VSMCs) were obtained from Invitrogen (Mountain View, CA). Cells were cultured as recommended by the manufacturer and used between passages 5 and 9.

### RNA Isolation and Quantitative RT-PCR

Total RNA was extracted from cells using RNeasy plus kit (Qiagen), per the manufacturer’s recommendations. Superscript II (Invitrogen) and random primers were used for reverse transcription. Message expression of human Nox4 (primer sequences: CTGGAGGAGCTGGCTCGCCAACGAAG and GTGATCATGAGGAATAGCACCACCACCATGCAG) was measured by amplification of human VSMC cDNA using the LightCycler (Roche) real-time thermocycler and SYBR green dye. Quantitative PCR data analysis was performed using the mak3 module of the qPCR software library in the R environment [20, 21].

### siRNA Transfection

For experiments using siRNA, human VSMCs were transfected with 25 nM small interfering RNA against Nox4 (siNox4#1; 5’-AAACTGAGGTACAGCTGGATG-3’, siNox4#2; 5’-CAGCATCTGTTCTTAACCTCA-3’), palladin (siPall; 5’-ATCAGTTGTACTGGACGGCTA-3’), MRTF-A (siMRTF-A; 5’-ATGGAGCTGGTGGAGAAGAAC-3’) or with the All-Star negative control siRNA (siNeg) using Lipofectamine RNAiMAX in OPTI-MEM. Cells were then cultured in serum-free medium for 24 hr before treatment.

### Western Blot and Immunoprecipitation

VSMCs were lysed in 1% Triton X-100 buffer (25 mM Hepes, 25 mM NaCl, 2.5 mM EDTA, 10 mM Na-pyrophosphate, 10 mM NaF, 0.1 mM Na_3_VO_4_, 1% Triton X-100, and protease inhibitors) for all experiments. Whole cell lysates were utilized for western blot and immunoprecipitation experiments, as described previously [4, 22]. Protein concentration was measured using the Bradford Assay. Proteins were separated using SDS-PAGE and transferred to nitrocellulose membranes, blocked, and incubated with appropriate primary antibodies. Proteins were detected by ECL (Amersham). Band intensity and migration was quantified by using Image J 1.46 and Carestream Molecular Imaging software, respectively. The apparent molecular weight of the bands was estimated using internal molecular weight standards.

### Alkaline Phosphatase Treatment

Cells were lysed in Hepes-Triton lysis buffer (Boston Bioproducts, Ashland, MA) (25 mM Hepes (pH 7.4), 150 mM NaCl, 5 mM EDTA, and 1% Triton-X 100) containing halt protease inhibitor EDTA-free 100x and halt phosphatase inhibitor 100x (Pierce, Rockford, IL). Lysates were either not treated or treated with 20 units of alkaline phosphatase (Fermentas) at 37°C for 30 min and subjected to western blot analysis.

### Statistical Analysis

Results are expressed as mean ± SE from at least three independent experiments unless otherwise indicated. Significance of statistical comparisons was assessed using one or two way analysis of variance (ANOVA), followed by Bonferroni post-hoc test when appropriate. A value of p<0.05 was considered significant. All data sets supporting each figure can be found in S1 Dataset.

## Results

### TGF-β-induced MRTF-A Phosphorylation is Redox-Sensitive and siNOX4 Blocks MRTF-A Phosphorylation and Expression

**Fig 1:**
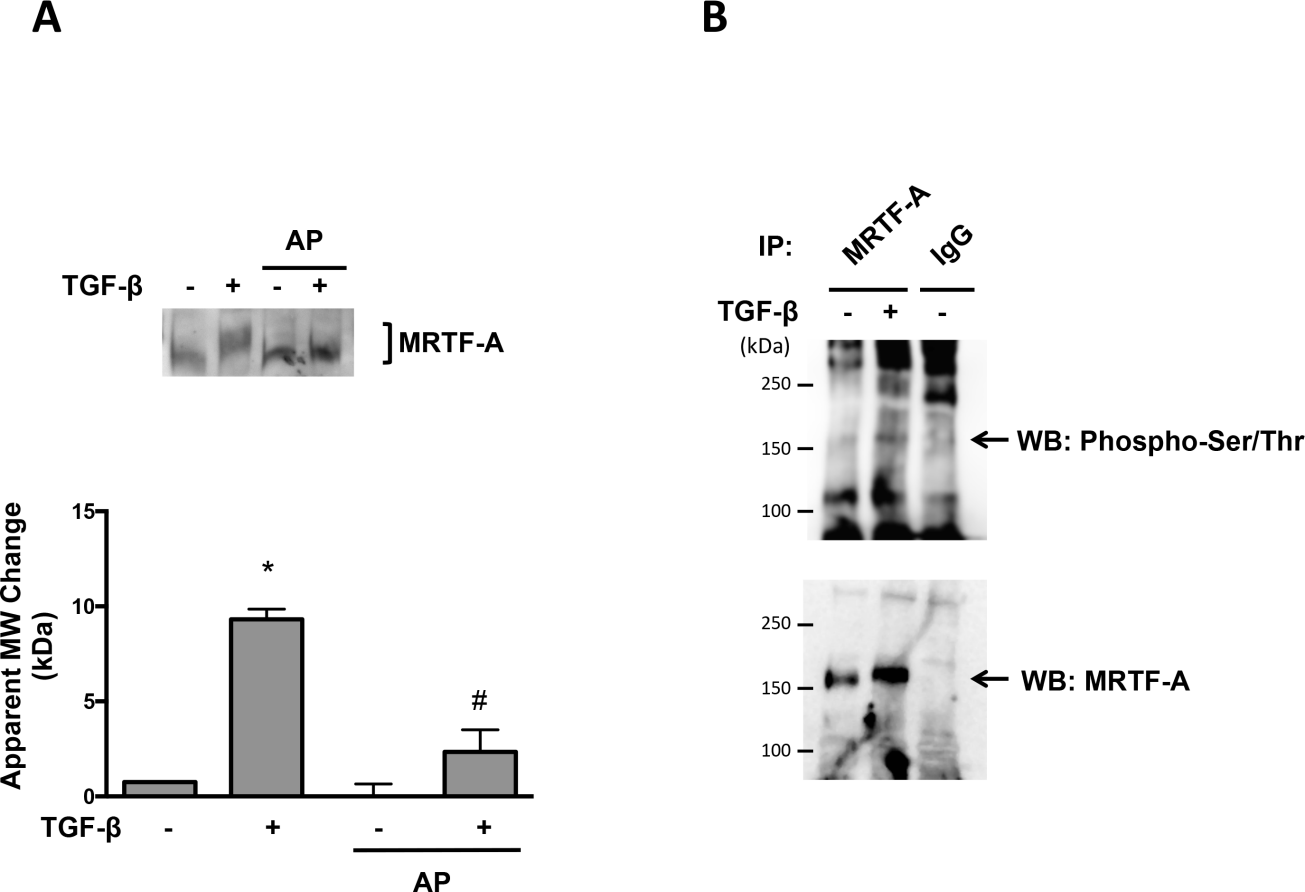
TGF-β-induced MRTF-A Molecular Weight Shift is Due To Phosphorylation. Human VSMCs were treated with TGF-β (2 ng/ml) for 24 hr. (A) Lysates were incubated with or without alkaline phosphatase at 37°C for 30 min. Western blot was performed using MRTF-A antibody. Bars represent mean ± SE of 5 independent experiments. *p<0.05 vs con and # p<0.05 vs TGF-β. (B) Total protein was extracted and immunoprecipitated with rabbit anti- MRTF-A antibody. Membranes were immunoblotted with phospho-Ser/Thr and goat anti-MRTF-A antibodies. This figure is representative of 3 independent experiments.

We first investigated the effect of TGF-β on MRTF-A phosphorylation in human aortic VSMCs. Although MRTF-A is known to be phosphorylated on multiple sites [9], there is little specific information and no available reagents to probe individual sites; therefore, we evaluated phosphorylation by measuring the upward shift in molecular weight, as has been done by others [9, 12]. As shown in Fig 1A, a 24-h treatment with TGF-β (2 ng/ml) induced a significant shift in molecular weight. Moreover, incubation of the lysate with alkaline phosphatase largely abolished the upward shift of MRTF-A in response to TGF-β, indicating that the molecular weight shift is due to phosphorylation of MRTF-A (Fig 1A). To confirm that the upward shift of MRTF-A is due to phosphorylation, we immunoprecipitated MRTF-A and blotted with phospho-Ser/Thr antibody (Fig 1B). The results show a predominant phospho-Ser/Thr band near 150 kDa that overlaps the MRTF-A band, supporting our conclusion that the molecular weight shift of MRTF-A is due to phosphorylation.

**Fig 2:**
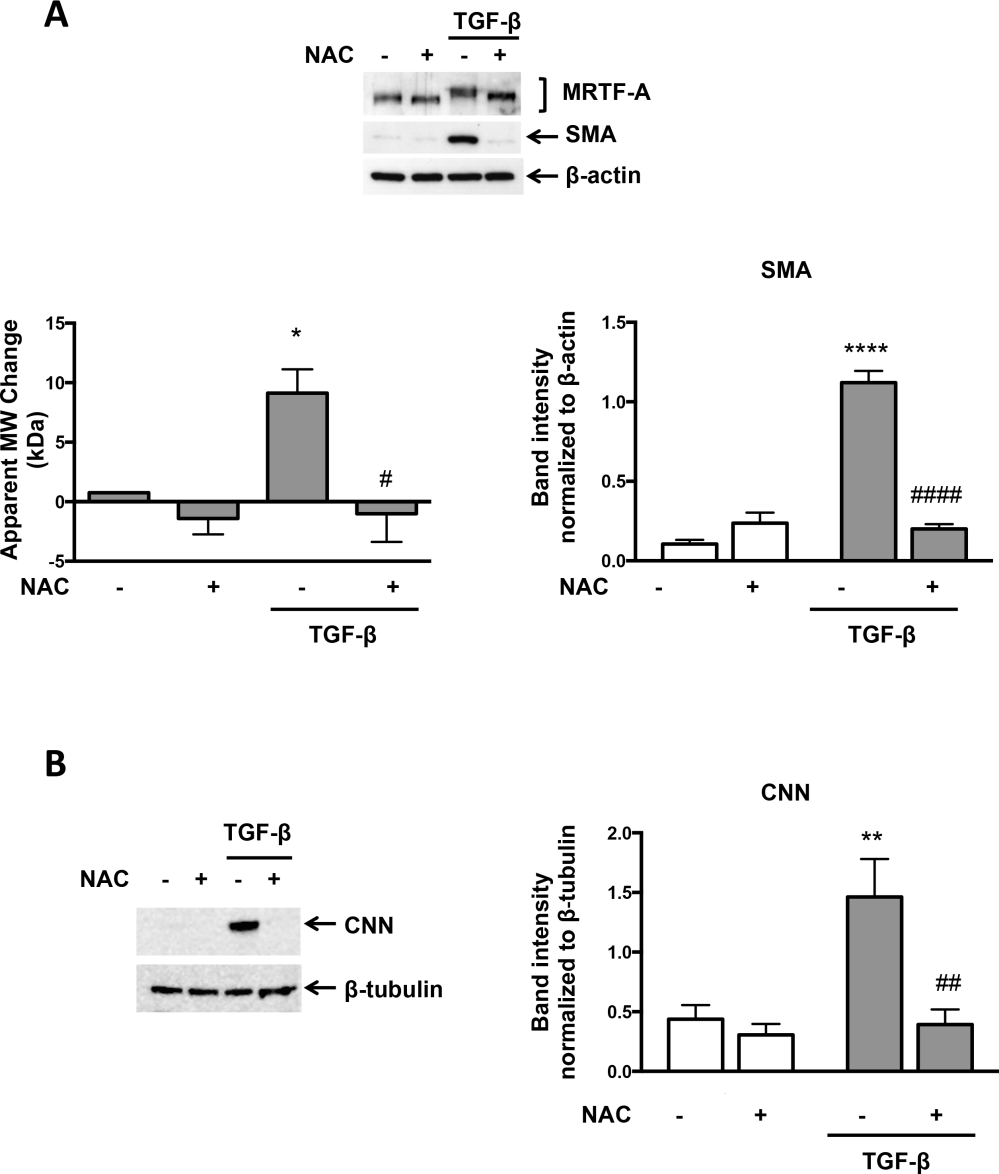
TGF-β-induced MRTF-A Phosphorylation and SMC Differentiation Marker Expression are Redox-Sensitive. Human VSMCs were preincubated with 20 mM NAC for 1 hr and then stimulated with TGF-β (2 ng/ml) for 24 hr. (A) Total protein was extracted and levels of MRTF-A and SMA were analyzed using specific antibodies. β-actin was used as a loading control. Bars represent mean ± SE of 3 independent experiments. *p<0.05 vs con, ****p<0.0001 vs con, # p<0.05 vs TGF-β and #### p<0.0001 vs TGF-β. (B) Total protein was extracted and the level of CNN was analyzed using a specific antibody. β-tubulin was used as a loading control. This figure is representative of 4 independent experiments. **p<0.01 vs con and ## p<0.01 vs TGF-β.

**Fig 3:**
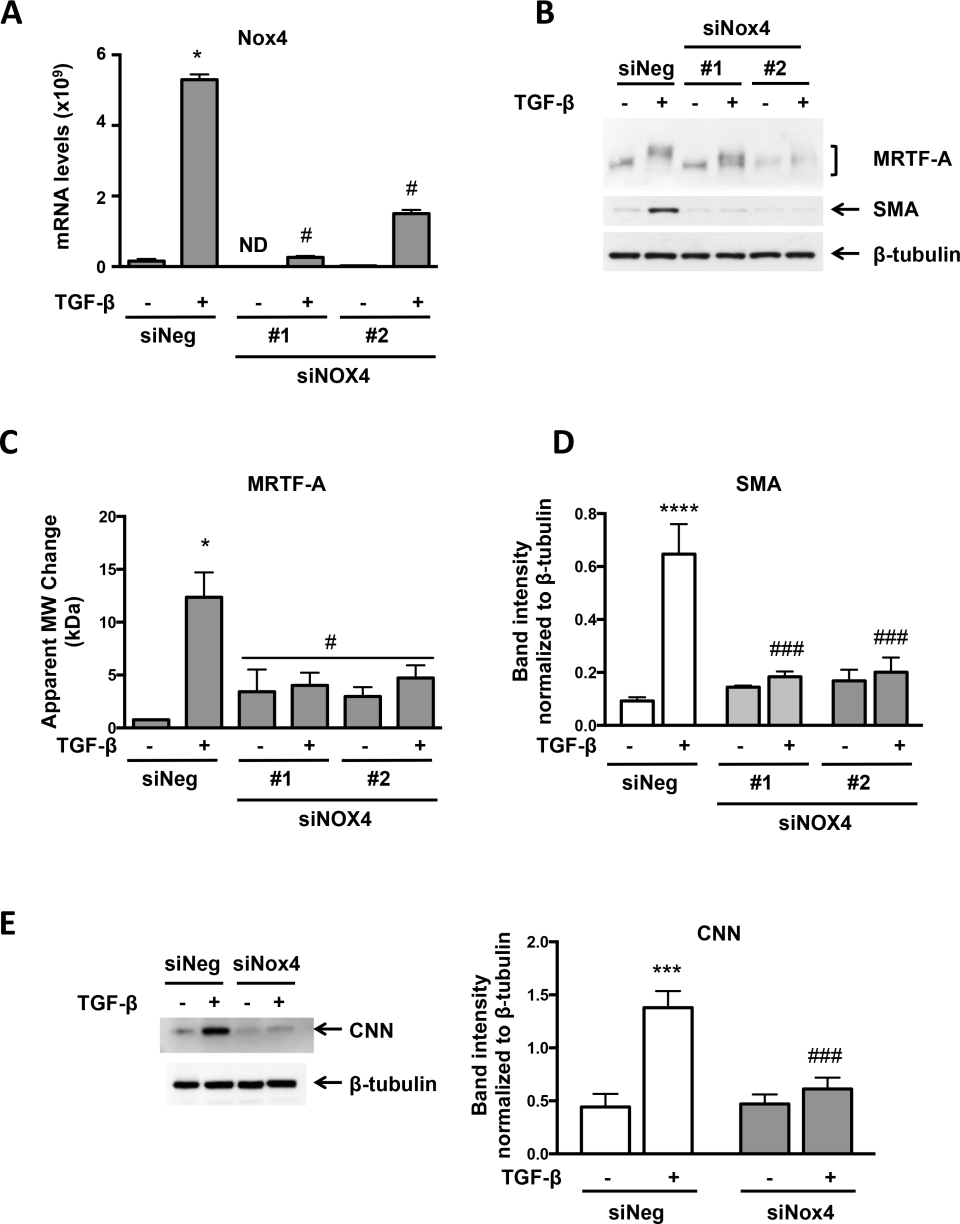
Nox4 is Necessary for TGF-β-induced MRTF-A Phosphorylation and SMC Differentiation Marker Expression. Human VSMCs were transfected with siRNA (siNeg) or siRNA against Nox4 (siNox4). Two different sequences for siNox4 were used. After 48 hr, the cells were treated with TGF-β (2 ng/ml) for 24 hr. (A) Nox4 mRNA was analyzed by real-time RT-PCR. Bars are means ± SE of 4 independent experiments. *p<0.05 vs siNeg, # p<0.05 vs siNeg + TGF-β. ND = not detectable. (B) Total protein was extracted and levels of MRTF-A and SMA were analyzed using specific antibodies. β-tubulin was used as a loading control. (C, D) Bars are means ± SE of 3 independent experiments. ****p<0.0001 vs siNeg, and ### p<0.001 vs TGF-β. E. siNo4 #2 was used for this experiment. Total protein was extracted and the level of CNN was analyzed using a specific antibody. β-tubulin was used as a loading control. Bars are means ± SE of 5 independent experiments. ***p<0.001 vs siNeg, ### p<0.001 vs siNeg + TGF-β.

To determine if TGF-β -induced MRTF-A phosphorylation requires ROS, VSMCs were treated with the antioxidant N-acetylcysteine (NAC) prior to TGF-β addition and then MRTF-A phosphorylation was evaluated. TGF-β- stimulated MRTF-A phosphorylation was abolished by NAC treatment (Fig 2A). Importantly, impaired MRTF-A phosphorylation correlated with inhibition of the expression of two of its target genes, SMA and CNN (Fig 2A and 2B). We have previously shown that 20 mM NAC completely scavenges NADPH oxidase-derived ROS in VSMCs [5, 13]. We therefore investigated if Nox4, the NADPH oxidase activated by TGF-β in these cells [5], affects MRTF-A phosphorylation. Using two different but effective siRNAs against Nox4 (Fig 3A), we found that knockdown of Nox4 reduced TGF-β-mediated MRTF-A phosphorylation and, in confirmation of our previous results, SMA and CNN expression as well (Fig 3B–3E).

### TGF-β-induced MRTF-A Phosphorylation is Partially Mediated by ROCK

**Fig 4:**
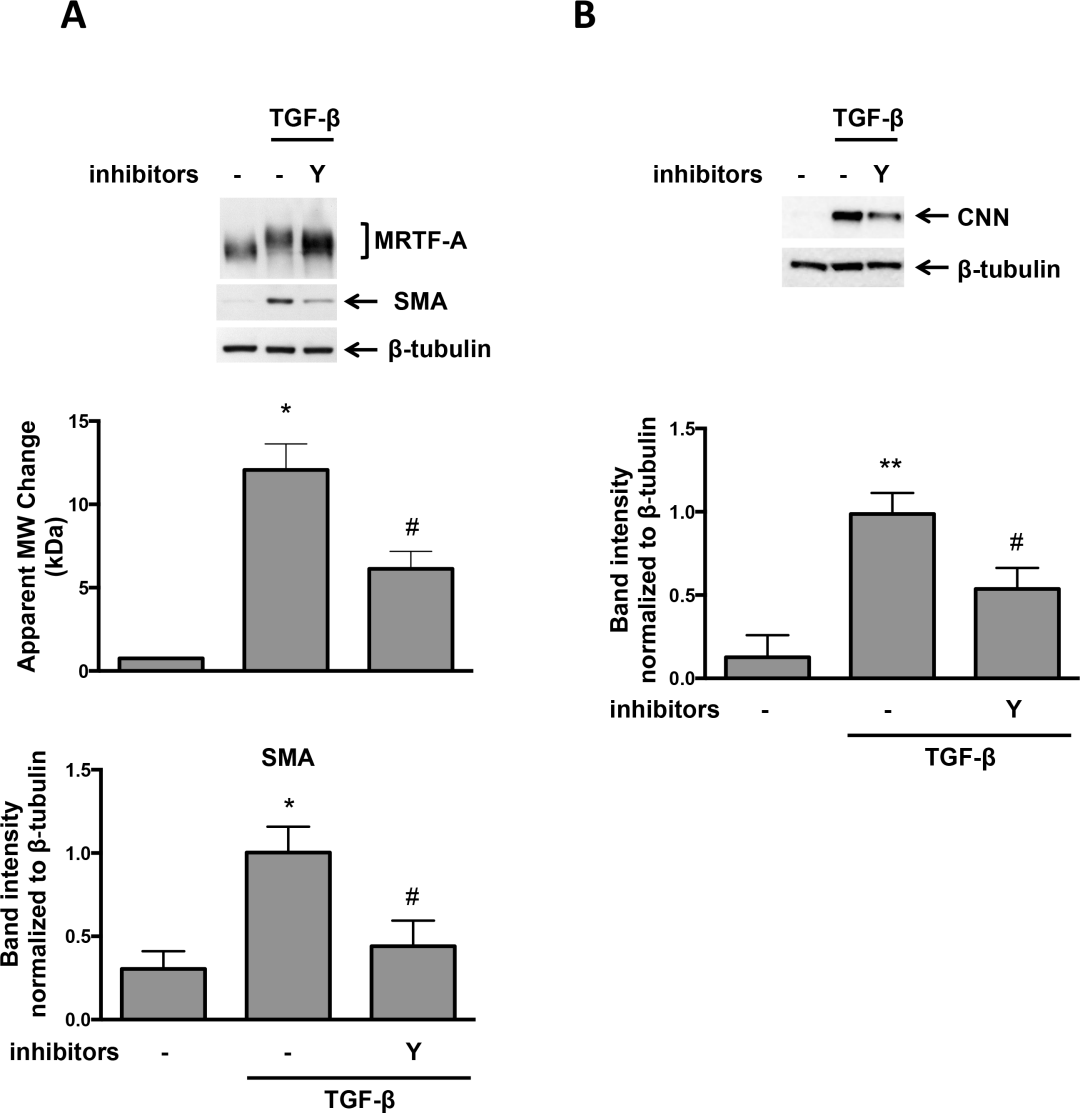
TGF-β-induced MRTF-A Phosphorylation is Partially Mediated by ROCK. Human VSMCs were preincubated with 10 μM ROCK inhibitor (Y-27632) for 1 hr. Then the cells were treated with TGF-β (2 ng/ml) for 24 hr. (A) Total protein was extracted and levels of MRTF-A was analyzed using specific antibody. β-tubulin was used as a loading control. Bars are means ± SE of 3 independent experiments. Y; Y-27632. *p<0.05 vs con and # p<0.05 vs TGF-β. (B) Total protein was extracted and levels of CNN was analyzed using specific antibody. β-tubulin was used as a loading control. Bars are means ± SE of 3 independent experiments. **p<0.01 vs con and # p<0.05 vs TGF-β.

As noted above, RhoA is downstream of Nox4 in VSMCs [13] and in NIH3T3 cells, C3 transferase abolishes MRTF-A phosphorylation in response to fetal calf serum [9]. These observations suggest a role for ROCK in MRTF-A phosphorylation, although this has not been tested in VSMCs or with TGF-β as the stimulus. We therefore used Y-27632, a specific ROCK inhibitor, to determine if ROCK mediates MRTF-A phosphorylation in response to TGF-β in VSMCs. Y-27632 treatment significantly reduced MRTF-A phosphorylation and SMA and CNN expression (Fig 4A and 4B), suggesting that in VSMCs, ROCK participates in MRTF-A phosphorylation after TGF-β treatment.

### Induction of Palladin by TGF-β is Redox-Sensitive and Its Expression Requires Nox4

**Fig 5:**
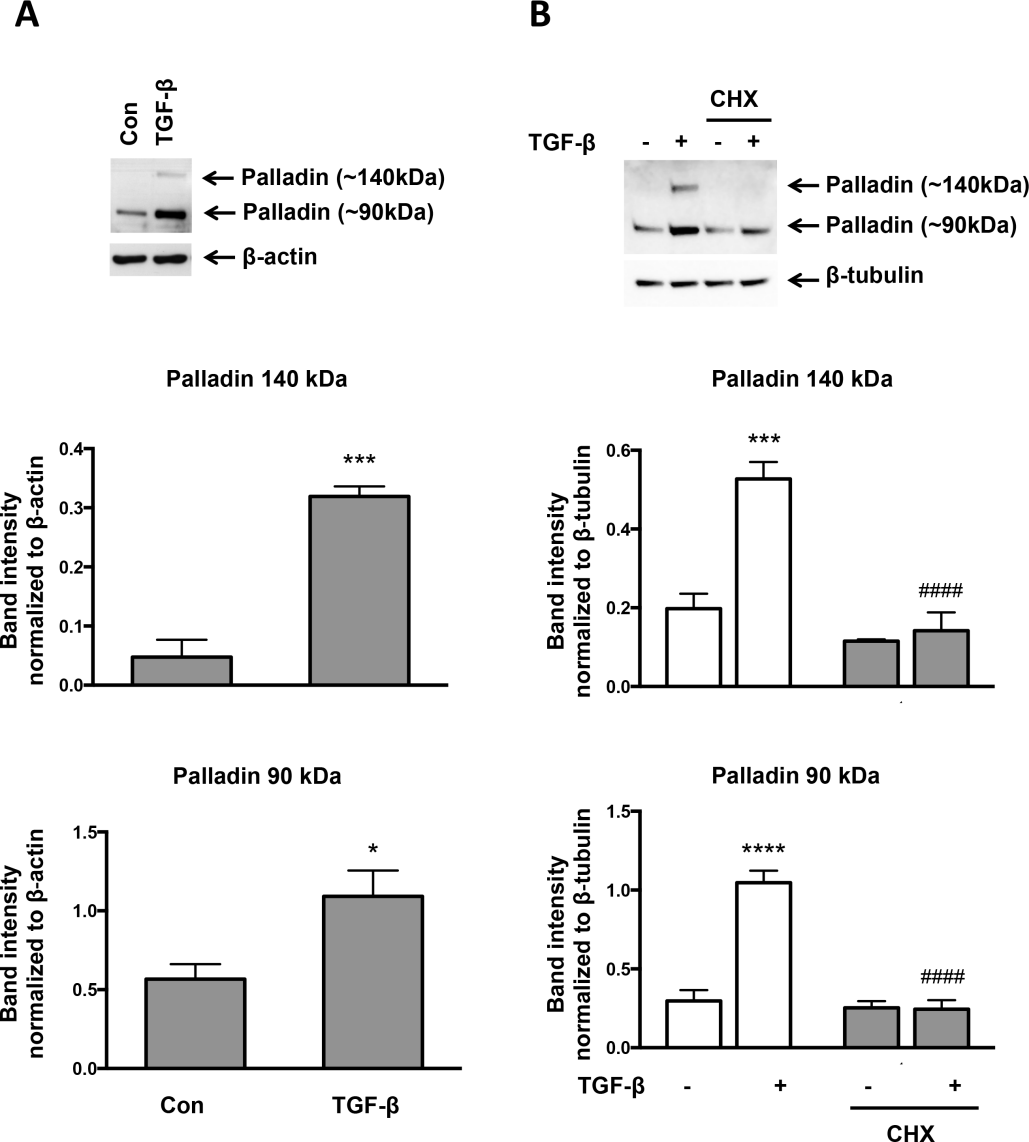
Palladin Expression is Induced by TGF-β. (A) Human VSMCs were incubated with TGF-β (2 ng/ml) for 24 hr. Total protein was extracted and the level of palladin was analyzed using a specific antibody. β-actin was used as a loading control. Bars are means ± SE of 3 independent experiments. *p<0.05 vs con and ***p<0.001 vs con. Con = control cells untreated with TGF-β. (B) Human VSMCs were preincubated with 100 μM cyclohexamide (CHX) for 30 min. Cells were then treated with TGF-β (2 ng/ml) for 24 hr. Total protein was extracted and levels of palladin were analyzed using a specific antibody. β-tubulin was used as a loading control. Bars are means ± SE of 4 independent experiments. ***p<0.001 vs con, ****p<0.0001 vs con, and #### p<0.0001 vs TGF-β.

**Fig 6:**
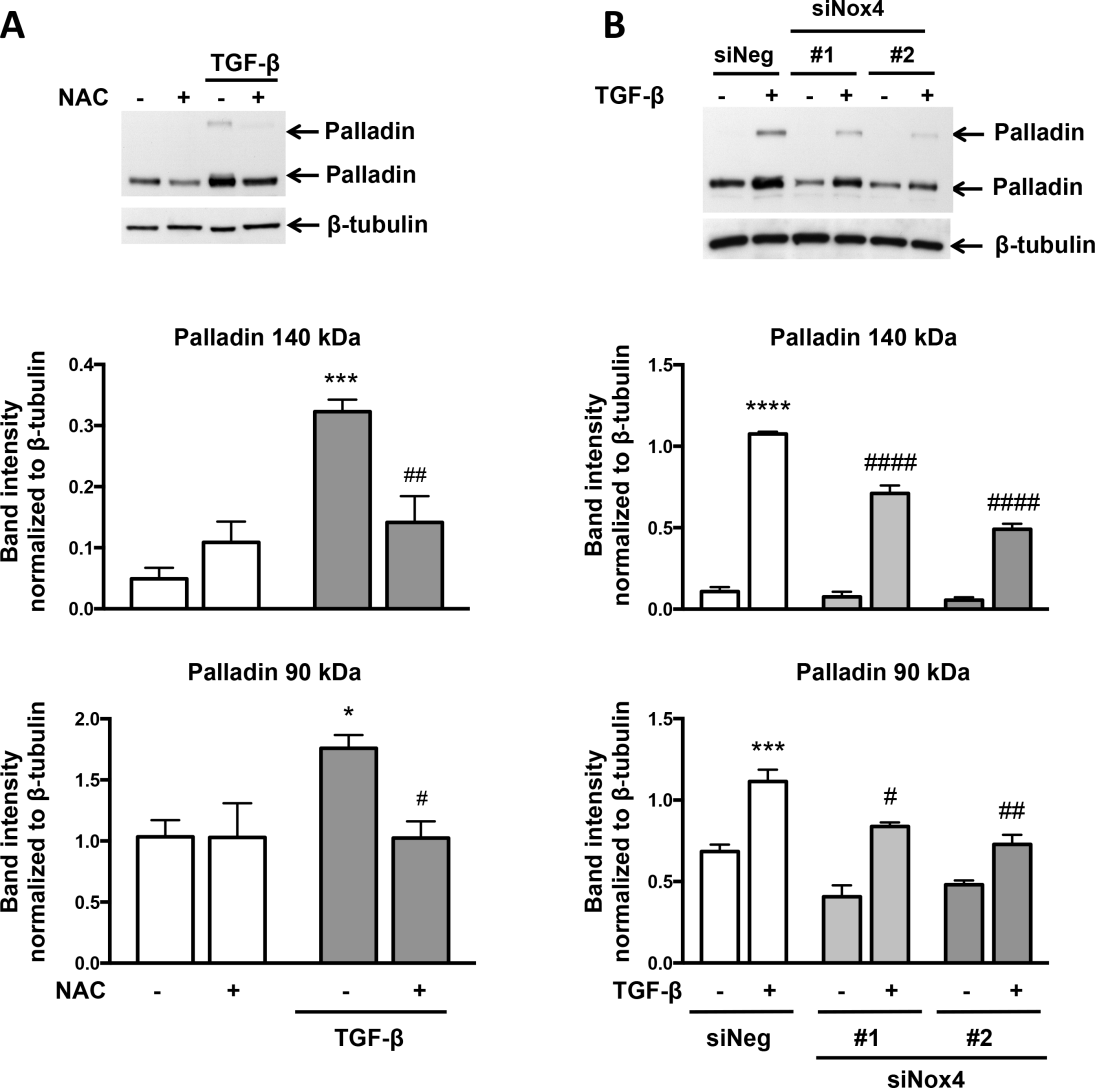
Induction of Palladin by TGF-β is Redox-Sensitive and Its Expression Requires Nox4. (A) Human VSMCs were preincubated with 20 mM NAC for 1 hr and then stimulated with TGF-β (2 ng/ml) for 24 hr. Total protein was extracted and levels of palladin were analyzed using a specific antibody. β-tubulin was used as a loading control. Bars are means ± SE of 3 independent experiments. *p<0.05 vs con, ***p<0.001 vs con, # p<0.05 vs TGF-β, and ## p<0.01 vs TGF-β. (B) Human VSMCs were transfected with control siRNA (siNeg) or siRNA against Nox4 (siNox4). Two different sequences for siNox4 were used. After 48 hr, the cells were treated with TGF-β (2 ng/ml) for 24 hr. Total protein was extracted and the level of palladin was analyzed using a specific antibody. β-tubulin was used as a loading control. Bars are means ± SE of 3 independent experiments. ***p<0.001 vs siNeg, ****p<0.0001 vs siNeg, # p<0.05 vs siNeg + TGF-β, ## p<0.01 vs siNeg + TGF-β, and #### p<0.0001 vs siNeg + TGF-β.

To explore a role for other potential Nox4 effectors in TGF-β-induced SMA and CNN expression, we focused on palladin because, like knockdown of Nox4, deletion of palladin results in a loss of actin fibers [23] and, like activation of Nox4, overexpression of palladin activates Rho and induces stress fibers [24]. We first determined whether TGF-β alters palladin expression in VSMCs. Cells were treated with TGF-β for 24 hr and analyzed by western blot. As shown in Fig 5A, TGF-β increased expression of the 90-kDa palladin isoform and caused neo-expression of the 140-kDa isoform of palladin. To determine if the effect of TGF-β on palladin is due to its increased expression or due to its decreased degradation, we treated cells with the protein synthesis inhibitor cyclohexamide (Fig 5B). Cyclohexamide treatment blocked TGF-β-induced palladin expression, suggesting that TGF-β increases palladin protein expression rather than decreasing its degradation. Treatment with NAC significantly reduced TGF-β-stimulated palladin expression in VSMCs (Fig 6A), as did knockdown of Nox4 using siRNA (Fig 6B), an effect that was accompanied by loss of SMA expression (Fig 3B). This effect on palladin appears to be independent of ROCK, because Y-27632 treatment had no effect on palladin expression (palladin 140 kDa; TGF-β 0.52 ± 0.06 vs TGF-β + inhibitor 0.58 ± 0.02, palladin 90 kDa; TGF-β 1.39 ± 0.04 vs TGF-β + inhibitor 1.27 ± 0.07).

### TGF-β-induced MRTF-A Phosphorylation and SMA and CNN Expression are Abolished in the Absence of Palladin

**Fig 7:**
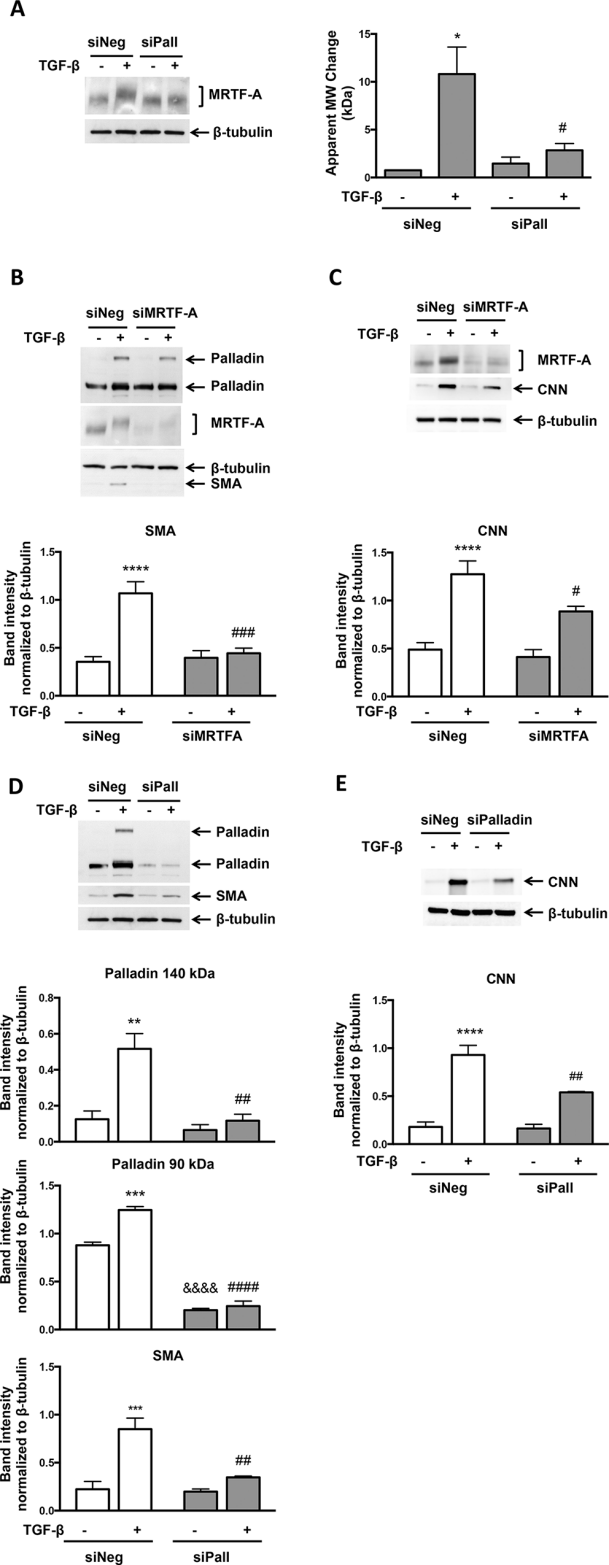
TGF-β-induced MRTF-A Phosphorylation and SMA Expression Require Palladin. (A) Human VSMCs were transfected with control siRNA (siNeg) or siRNA against palladin (siPall). After 48 hr, the cells were treated with TGF-β (2 ng/ml) for 24 hr. Total protein was extracted and levels of MRTF-A were analyzed using a specific antibody. β-tubulin was used as a loading control. Bars are means ± SE of 3 independent experiments. *p<0.05 vs siNeg and # p<0.05 vs siNeg + TGF-β. (B,C) Human VSMCs were transfected with control siRNA (siNeg) or siRNA against MRTF-A (siMRTF-A) and treated as in A. Total protein was extracted and levels of palladin, SMA, and CNN were analyzed using specific antibodies. β-tubulin was used as a loading control. Bars (B) are means ± SE of 4 independent experiments. Bars (C) are means ± SE of 3 independent experiments. ****p<0.0001 vs siNeg, # p<0.05 vs siNeg + TGF-β, and ### p<0.001 vs siNeg + TGF-β. (D) Human VSMCs were treated as in A. Bars are means ± SE of 3 independent experiments. *p<0.05 vs siNeg, **p<0.01 vs siNeg, ## p<0.01 vs siNeg + TGF-β, #### p<0.0001 vs siNeg + TGF-β, and &&&& p<0.0001 vs siNeg. (E) Human VSMCs were transfected with control siRNA (siNeg) or siRNA against palladin (siPall). After 48 hr, the cells were treated with TGF-β (2 ng/ml) for 48 hr. Total protein was extracted and levels of CNN were analyzed using a specific antibody. β-tubulin was used as a loading control. Bars are means ± SE of 4 independent experiments. ****p<0.0001 vs siNeg, ## p<0.01 vs siNeg + TGF-β.

Jin *et al*. [14] showed that palladin binds to MRTF-A, but the function of this interaction remains unclear. Based on this finding and considering that palladin expression is controlled by TGF-β/Nox4 in VSMC, we tested if knockdown of palladin affects TGF-β-induced MRTF-A phosphorylation. Indeed, knockdown of palladin prevented TGF-β-stimulated MRTF-A phosphorylation (Fig 7A).

To test whether palladin could also act downstream of MRTF-A, siMRTF-A was transfected into VSMCs and palladin expression was measured. siMRTF-A transfection did not change palladin expression (Fig 7B), even though MRTF-A levels were nearly abolished. This suggests that palladin is upstream of MRTF-A phosphorylation. As expected, TGF-β-induced SMA and CNN expression was decreased by siMRTF-A (Fig 7B and 7C), confirming that MRTF-A has a key role in regulating SMA and CNN expression. Taken together, these results imply that palladin acts upstream of TGF-β-induced MRTF-A phosphorylation.

Finally, to determine if TGF-β-induced SMA expression requires palladin, siRNA against palladin was transfected into human VSMCs. Knockdown of both forms of palladin was confirmed using palladin antibody, and led to decreased SMA and CNN expression (Fig 7D and 7E). Thus, palladin is required for TGF-β-induced SMA and CNN expression.

## Discussion

**Fig 8:**
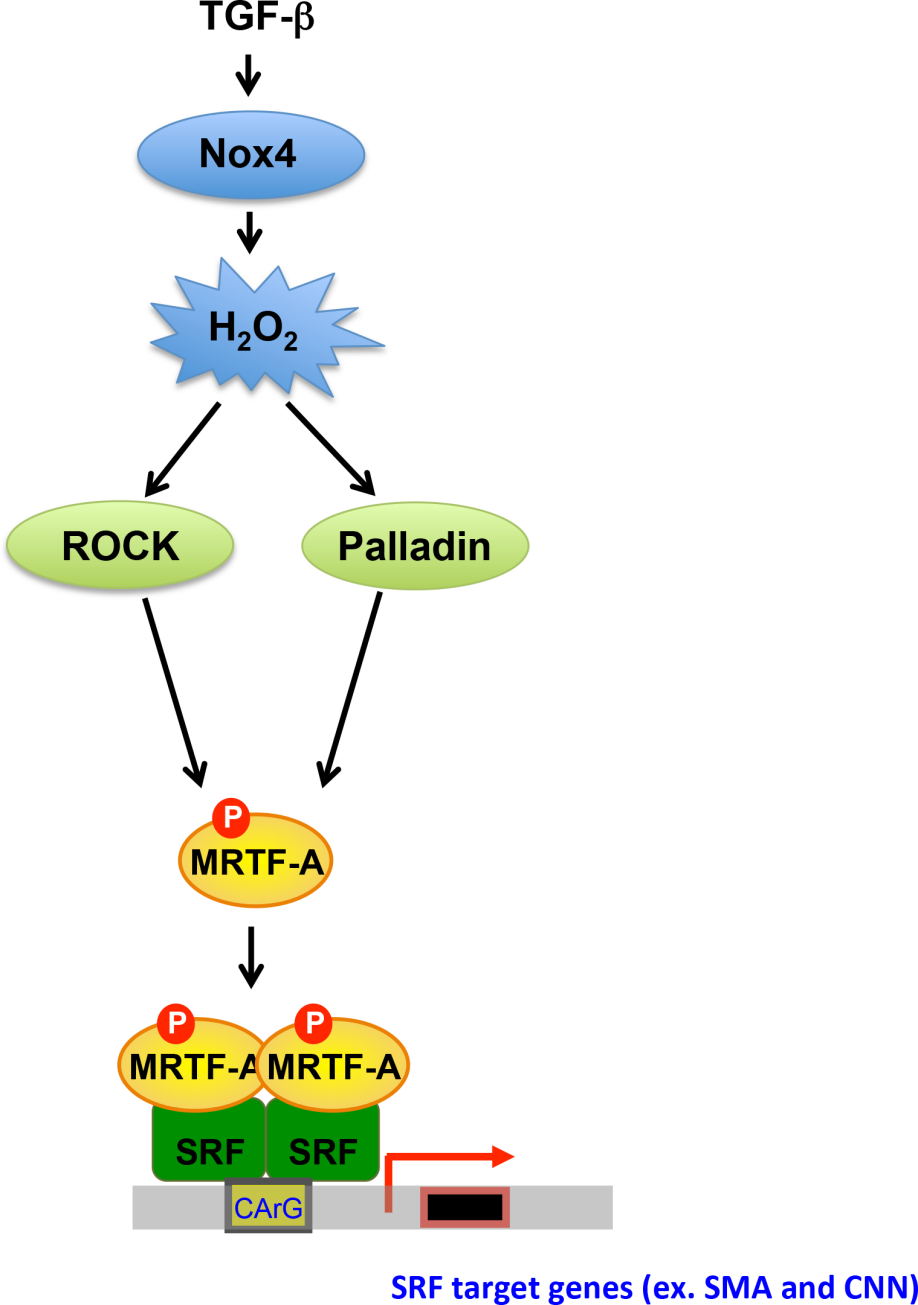
Proposed Model of Redox-Sensitive Regulation of MRTF-A Phosphorylation via Palladin in Human VSMCs. Stimulation of Human VSMCs with TGF-β (2 ng/ml) upregulates Nox4, increasing H_2_O_2_ production. H_2_O_2_ increases expression of palladin and stimulates the activity of ROCK, which phosphorylates MRTF-A. MRTF-A binds to SRF which induces SMA and CNN expression.

In this study, we explored the relationship between Nox4, palladin, ROCK, MRTF-A and regulation of SMC-specific gene expression. We demonstrated that 1) TGF-β-induced phosphorylation of MRTF-A is mediated by Nox4 via ROCK and palladin; 2) TGF-β induction of palladin is redox-sensitive and requires ROS production from Nox4; and 3) knockdown of palladin, reduction in Nox4 activity or inhibition of ROCK attenuates SMA and CNN expression. Our data uncover a previously unknown mechanism for how Nox4 regulates SMA and CNN expression; that is, via regulation of palladin expression and ROCK-mediated MRTF-A phosphorylation (Fig 8).

Palladin has multiple isoforms created by alternative transcription initiation and splicing that are differentially expressed in cells and tissues [25]. The 90-kDa form of palladin was previously reported to be upregulated and the 140-kDa isoform of palladin to be neo-expressed by TGF-β during fibroblast-to-myofibroblast conversion [26]. Palladin has several functions in actin-based subcellular structures [16], acting as a molecular scaffold and actin cross linker [27–29]. In addition, recent findings suggested its involvement in transcriptional regulation, showing that palladin is essential for SMC marker gene expression [14, 15]. Our results agree with these previous reports, and extend these observations by demonstrating a role for Nox4 in palladin-dependent SMA and CNN expression.

Previously, hydrogen peroxide was used to see if ROS alter palladin expression in SMCs [30]. The authors found that extracellular application of 100 μM H_2_O_2_ decreased palladin expression after 24 hr of treatment. In contrast, we found that the intracellular production of likely smaller amounts of H_2_O_2_ by Nox4 activation [5] is actually required for TGF-β-induced palladin expression. The discrepancy between these studies most likely relates to the lack of specificity of extracellularly applied H_2_O_2_, which easily diffuses through membranes, is not locally produced and is applied in amounts in great excess of those produced locally by NADPH oxidases. The involvement of Nox4 in palladin expression is consistent with the role of this oxidase in maintenance of differentiation and in regulation of cytoskeletal dynamics [1, 4, 13].

How palladin participates in transcriptional regulation is not clear; however, palladin was found to bind MRTF-A in the nucleus in VSMCs [14], raising the possibility that palladin may have a role in regulation of MRTF-A activity for SMC marker gene expression. Our results show that both palladin and MRTF-A are under the regulation of Nox4-derived ROS, suggesting possible cross-talk in the signaling. Knockdown of palladin using siRNA abolishes MRTF-A phosphorylation but not expression, which indicates that palladin is upstream of MRTF-A phosphorylation, an idea consistent with the fact that knockdown of MRTF-A did not affect the expression levels of palladin. Since palladin does not have kinase activity, it is likely that ROCK is one of the downstream kinases that mediate MRTF-A phosphorylation. However, the large molecular weight change of MRTF-A in response to TGF-β suggests that MRTF-A is phosphorylated at multiple sites, which strongly suggests that additional kinases, dependent or independent of Nox4, phosphorylate MRTF-A. It is worth noting that MRTF-B may also be a target of Nox4/ROCK because it possesses similar domains and interacts with SRF in a similar manner to MRTF-A [31, 32].

Based on previous data and the data contained herein, the role of Nox4/palladin in MRTF-A phosphorylation can be narrowed down to five potential possibilities. First, palladin may act to regulate ROCK activity. Therefore, the effect of palladin on ROCK activity should be tested in the future. Second, palladin may help to localize MRTF-A to the nucleus. There is currently no evidence that knockdown of palladin changes MRTF-A’s subcellular localization, but this possibility must be rigorously tested. Third, palladin may regulate phosphatase activity. It is possible that the MRTF-A phosphatase is inhibited by palladin, allowing MRTF-A to remain phosphorylated for up to 24 hr. Clearly, additional studies will be required to explore this hypothesis. Fourth, palladin may dissociate monomeric actin and MRTF-A binding by facilitating actin polymerization and bundling [29, 33, 34], a process that can be affected by actin oxidation [7]. Jin et al. [15] showed a decreased F-actin to G-actin ratio in palladin-null SMCs differentiating from embryoid bodies, which suggests a role for palladin in providing G-actin to sequester MRTF-A. Finally, palladin may regulate MRTF-A phosphorylation as a parallel pathway to Nox4. Since knockdown Nox4 decreased TGF-β-induced palladin expression, it is likely to be under the control of Nox4. However, our attempts to knockdown Nox4 and overexpress palladin in human aortic smooth muscle cells proved technically difficult and the possibility of parallel pathways should be tested in the future. Further work will be necessary to dissect the precise relationships among Nox4, palladin and MRTF-A.

In conclusion, these data demonstrate that ROCK-mediated MRTF-A phosphorylation is crucial in regulation of SRF responsive genes in VSMCs and that this event is regulated by Nox4-mediated palladin expression. These findings may contribute to the discovery of new targets for prevention and recovery from cardiovascular diseases such as atherosclerosis and in-stent restenosis.

## Supporting Information

S1 Dataset **Data underlying each Fig F1 dataset, Data underlying Fig 1A.** Molecular weight change of MRTF-A; **F2 Dataset, Data underlying Fig 2.** (A) Data underlying Fig 2A. Molecular weight change of MRTF-A, (B) Data underlying Fig 2A. Ratio SMA/beta-actin, (C) Data underlying Fig 2B. Ratio CNN/Tubulin**; F3 Dataset, Data underlying Fig 3.** (A) Data underlying Fig 3A. Nox4 mRNA level, (B) Data underlying Fig 3C. Molecular weight change of MRTF-A, (C) Data underlying Fig 3D. Ratio SMA/Tubulin, (D) Data underlying Fig 3E. Ratio CNN/Tubulin**; F4 Dataset, Data underlying Fig 4.** (A) Data underlying Fig 4A. Molecular weight change of MRTF-A, (B) Data underlying Fig 4A. Ratio SMA/Tubulin, (C) Data underlying Fig 4B. Ratio CNN/Tubulin; **F5 Dataset, Data underlying Fig 5.** (A) Data underlying Fig 5A. Ratio Palladin 140 kDa/Tubulin, (B) Data underlying Fig 5A. Ratio Palladin 90 kDa/Tubulin, (C) Data underlying Fig 5B. Ratio Palladin 140 kDa/Tubulin, (D) Data underlying Fig 5B. Ratio Palladin 90 kDa/Tubulin**; F6 Dataset, Data underlying Fig 6.** (A) Data underlying Fig 6A. Ratio Palladin 140 kDa/Tubulin, (B) Data underlying Fig 6A. Ratio Palladin 90 kDa/Tubulin, (C) Data underlying Fig 6B. Ratio Palladin 140 kDa/Tubulin, (D) Data underlying Fig 6B. Ratio Palladin 90 kDa/Tubulin**; F7 Dataset, Data underlying Fig 7.** (A) Data underlying Fig 7A. Molecular weight of MRTF-A, (B) Data underlying Fig 7B. Ratio SMA/Tubulin, (C) Data underlying Fig 7C. Ratio CNN/Tubulin, (D) Data underlying Fig 7D. Ratio Palladin 140 kDa/Tubulin, (E) Data underlying Fig 7D. Ratio Palladin 90 kDa/Tubulin, (F) Data underlying Fig 7D. Ratio SMA/Tubulin., (G) Data underlying Fig 7E. Ratio CNN/Tubulin.
(PDF)
