# Supplementary material for: Redox-Sensitive Regulation of Myocardin-Related Transcription Factor (MRTF-A) Phosphorylation via Palladin in Vascular Smooth Muscle Cell Differentiation Marker Gene Expression
Source: PLoS One. 2016 Apr 18;11(4):e0153199. doi: 10.1371/journal.pone.0153199 (PMC4835087; doi:10.1371/journal.pone.0153199)
Supplement: S1 Dataset — Data underlying each Fig F1 dataset, Data underlying Fig 1A. Molecular weight change of MRTF-A; F2 Dataset, Data underlying Fig 2. (A) Data underlying Fig 2A. Molecular weight change of MRTF-A, (B) Data underlying Fig 2A. Ratio SMA/beta-actin, (C) Data underlying Fig 2B. Ratio CNN/Tubulin; F3 Dataset, Data underlying Fig 3. (A) Data underlying Fig 3A. Nox4 mRNA level, (B) Data underlying Fig 3C. Molecular weight change of MRTF-A, (C) Data underlying Fig 3D. Ratio SMA/Tubulin, (D) Data underlying Fig 3E. Ratio CNN/Tubulin; F4 Dataset, Data underlying Fig 4. (A) Data underlying Fig 4A. Molecular weight change of MRTF-A, (B) Data underlying Fig 4A. Ratio SMA/Tubulin, (C) Data underlying Fig 4B. Ratio CNN/Tubulin; F5 Dataset, Data underlying Fig 5. (A) Data underlying Fig 5A. Ratio Palladin 140 kDa/Tubulin, (B) Data underlying Fig 5A. Ratio Palladin 90 kDa/Tubulin, (C) Data underlying Fig 5B. Ratio Palladin 140 kDa/Tubulin, (D) Data underlying Fig 5B. Ratio Palladin 90 kDa/Tubulin; F6 Dataset, Data underlying Fig 6. (A) Data underlying Fig 6A. Ratio Palladin 140 kDa/Tubulin, (B) Data underlying Fig 6A. Ratio Palladin 90 kDa/Tubulin, (C) Data underlying Fig 6B. Ratio Palladin 140 kDa/Tubulin, (D) Data underlying Fig 6B. Ratio Palladin 90 kDa/Tubulin; F7 Dataset, Data underlying Fig 7. (A) Data underlying Fig 7A. Molecular weight of MRTF-A, (B) Data underlying Fig 7B. Ratio SMA/Tubulin, (C) Data underlying Fig 7C. Ratio CNN/Tubulin, (D) Data underlying Fig 7D. Ratio Palladin 140 kDa/Tubulin, (E) Data underlying Fig 7D. Ratio Palladin 90 kDa/Tubulin, (F) Data underlying Fig 7D. Ratio SMA/Tubulin., (G) Data underlying Fig 7E. Ratio CNN/Tubulin. (PDF) [file pone.0153199.s001.pdf]

S1 Dataset, Data underlying each figure.

F1 Dataset, Data underlying Fig 1A.

Molecular weight change of MRTF-A.

Samples	Exp 1	Exp 2	Exp 3	n	m	SEM
Cont	0.7600	0.7600	0.7600	3	0.7600	0.0000
NAC	8.2505	9.9967	9.7158	3	9.3210	0.5413
TGF-β	0.7600	0.5526	-1.3068	3	0.0019	0.6571
TGF-β + NAC	1.4105	0.9678	4.6600	3	2.3461	1.1640

F2 Dataset, Data underlying Fig 2.

(A) Data underlying Fig 2A. Molecular weight change of MRTF-A.

Samples	Exp 1	Exp 2	Exp 3	n	m	SEM
Cont	0.7500	0.7600	0.7600	3	0.7567	0.0033
NAC	-2.9977	-2.4833	1.2521	3	-1.4096	1.3391
TGF-β	6.5541	7.7189	13.0800	3	9.1177	2.0095
TGF-β + NAC	-1.6023	3.3601	-4.7894	3	-1.0105	2.3711

(B) Data underlying Fig 2A. Ratio SMA/beta-actin.

Samples	Exp 1	Exp 2	Exp 3	n	m	SEM
Cont	0.1582	0.0763	0.0805	3	0.1050	0.0266
NAC	0.1766	0.3692	0.1658	3	0.2372	0.0661
TGF-β	1.0731	1.0226	1.2646	3	1.1201	0.0737
TGF-β + NAC	0.2548	0.1947	0.1518	3	0.2005	0.0299

(C) Data underlying Fig 2B. Ratio CNN/Tubulin.

Samples	Exp 1	Exp 2	Exp 3	Exp 4	n	m	SEM
Cont	0.0804	0.5998	0.5313	0.5356	4	0.4368	0.1198
NAC	0.0749	0.2412	0.4611	0.4460	4	0.3058	0.0919
TGF-β	2.3604	1.4429	0.9333	1.1156	4	1.4630	0.3171
TGF-β + NAC	0.0818	0.3136	0.6717	0.5002	4	0.3918	0.1266

F3 Dataset, Data underlying Fig 3.

(A) Data underlying Fig 3A. Nox4 mRNA level.

Samples	Exp 1	Exp 2	Exp 3	n	m	SEM
**siNeg**	2.0126E-10	1.6633E-10	8.6908E-11	3	1.5150E-10	3.3832E-11
**siNeg + TGF-β**	5.3984E-09	5.1228E-09	5.3711E-09	3	5.2974E-09	8.7659E-11
**siNox4B#1**	7.1728E-12	5.4823E-14	6.7697E-13	3	2.6349E-12	2.2761E-12
**siNox4B#1 + TGF-β**	2.2250E-10	2.6629E-10	2.9959E-10	3	2.6279E-10	2.2324E-11
**siNox4#2**	2.5450E-11	2.3494E-11	1.7177E-11	3	2.2040E-11	2.4964E-12
**siNox4#2 + TGF-β**	1.4273E-09	1.6249E-09	1.4542E-09	3	1.5021E-09	6.1857E-11

(B) Data underlying Fig 3C. Molecular weight change of MRTF-A.

Samples	Exp 1	Exp 2	Exp 3	n	m	SEM
**siNeg**	0.7602	0.7879	0.7600	3	0.7694	0.0093
**siNeg + TGF-β**	8.1525	12.6221	16.2777	3	12.3508	2.3495
**siNox4B#1**	1.9246	0.7879	7.5737	3	3.4288	2.0983
**siNox4B#1 + TGF-β**	1.6909	5.7437	4.6158	3	4.0168	1.2077
**siNox4#2**	1.2248	4.0712	3.6411	3	2.9790	0.8859
**siNox4#2 + TGF-β**	2.8656	6.9492	4.3703	3	4.7284	1.1923

(C) Data underlying Fig 3D. Ratio SMA/Tubulin.

Samples	Exp 1	Exp 2	Exp 3	n	m	SEM
**siNeg**	0.12116	0.07620	0.07881	3	0.0921	0.0146
**siNeg + TGF-β**	0.80107	0.71449	0.42560	3	0.6471	0.1135
**siNox4B#1**	0.13471	0.14238	0.15585	3	0.1443	0.0062
**siNox4B#1 + TGF-β**	0.15140	0.17718	0.22155	3	0.1834	0.0205
**siNox4#2**	0.11224	0.14271	0.25020	3	0.1684	0.0418
**siNox4#2 + TGF-β**	0.11561	0.18322	0.30418	3	0.2010	0.0552

(D) Data underlying Fig 3E. Ratio CNN/Tubulin.

Samples	Exp 1	Exp 2	Exp 3	Exp 4	Exp 5	n	m	SEM
**siNeg**	0.8049	0.0821	0.2839	0.4913	0.5502	5	0.4425	0.1225
**siNeg + TGF-β**	0.9898	1.6504	1.8302	1.1702	1.2574	5	1.3796	0.1561
**siNox4**	0.5894	0.1523	0.3904	0.6622	0.5581	5	0.4705	0.0912
**siNox4 + TGF-β**	0.5215	1.0206	0.4009	0.5818	0.5398	5	0.6129	0.1063

F4 Dataset, Data underlying Fig 4.

(A) Data underlying Fig 4A. Molecular weight change of MRTF-A.

Samples	Exp 1	Exp 2	Exp 3	Exp 4	n	m	SEM
**Cont**	0.7600	0.7600	0.7600	0.7600	4	0.7600	0.0000
**TGF-β**	15.2459	12.1260	6.4133	12.0391	4	1.0025	1.8389
**TGF-β + Y**	8.6814	6.2736	3.5575	6.0218	4	0.4404	1.0472

(B) Data underlying Fig 4A. Ratio SMA/Tubulin.

Samples	Exp 1	Exp 2	Exp 3	n	m	SEM
**Cont**	0.0944	0.3829	0.4359	3	0.3044	0.1061
**TGF-β**	1.2340	1.0666	0.7070	3	1.0025	0.1555
**TGF-β + Y**	0.4189	0.2980	0.6044	3	0.4404	0.0891

(C) Data underlying Fig 4B. Ratio CNN/Tubulin.

Samples	Exp 1	Exp 2	Exp 3	n	m	SEM
**Cont**	0.0744	0.2529	0.0316	3	0.1196	0.0678
**TGF-β**	0.9413	1.0293	0.9616	3	0.9774	0.0266
**TGF-β + Y**	0.6229	0.3564	0.6454	3	0.5415	0.0928

F5 Dataset, Data underlying Fig 5.

(A) Data underlying Fig 5A. Ratio Palladin 140 kDa/Tubulin.

Samples	Exp 1	Exp 2	Exp 3	n	m	SEM
**Cont**	0.0361	0.0809	0.0258	3	0.0476	0.0169
**TGF-β**	0.3306	0.2858	0.3409	3	0.3191	0.0169

(B) Data underlying Fig 5A. Ratio Palladin 90 kDa/Tubulin.

Samples	Exp 1	Exp 2	Exp 3	n	m	SEM
**Cont**	0.5786	0.3976	0.7259	3	0.5674	0.0949
**TGF-β**	1.0800	1.2610	0.9328	3	1.0913	0.0949

(C) Data underlying Fig 5B. Ratio Palladin 140 kDa/Tubulin.

Samples	Exp 1	Exp 2	Exp 3	Exp 4	n	m	SEM
**Cont**	0.1295	0.1936	0.3049	0.1626	4	0.1977	0.0381
**TGF-β**	0.6501	0.4878	0.5198	0.4505	4	0.5270	0.0434
**CHX**	0.1016	0.1232	0.1193	0.1165	4	0.1152	0.0047
**CHX + TGF-β**	0.1006	0.1772	0.0378	0.2522	4	0.1420	0.0465

(D) Data underlying Fig 5B. Ratio Palladin 90 kDa/Tubulin.

Samples	Exp 1	Exp 2	Exp 3	Exp 4	n	m	SEM
**Cont**	0.2829	0.3891	0.4078	0.1089	4	0.2972	0.0685
**TGF-β**	0.9931	0.8698	1.2288	1.0947	4	1.0466	0.0762
**CHX**	0.2941	0.2965	0.1248	0.2971	4	0.2531	0.0428
**CHX + TGF-β**	0.2715	0.2862	0.0801	0.3409	4	0.2447	0.0568

F6 Dataset, Underlying Fig 6.

(A) Data underlying Fig 6A. Ratio Palladin 140 kDa/Tubulin.

Samples	Exp 1	Exp 2	Exp 3	n	m	SEM
**Cont**	0.0395	0.0842	0.0233	3	0.0490	0.0182
**NAC**	0.0711	0.1770	0.0784	3	0.1088	0.0341
**TGF-β**	0.3620	0.2978	0.3083	3	0.3227	0.0199
**TGF-β + NAC**	0.1494	0.0630	0.2120	3	0.1415	0.0432

(B) Data underlying Fig 6A. Ratio Palladin 90 kDa/Tubulin.

Samples	Exp 1	Exp 2	Exp 3	n	m	SEM
**Cont**	1.0501	0.7878	1.2623	3	1.0334	0.1372
**NAC**	0.6322	1.5696	0.8858	3	1.0292	0.2799
**TGF-β**	1.9601	1.7321	1.5869	3	1.7597	0.1086
**TGF-β + NAC**	1.2044	0.7573	1.1118	3	1.0245	0.1362

(C) Data underlying Fig 6B. Ratio Palladin 140 kDa/Tubulin.

Samples	Exp 1	Exp 2	Exp 3	n	m	SEM
**siNeg**	0.1624	0.0765	0.0854	3	0.1081	0.0273
**siNeg + TGF-β**	1.0642	1.0601	1.1029	3	1.0757	0.0136
**siNox4B#1**	0.1336	0.0655	0.0283	3	0.0758	0.0308
**siNox4B#1 + TGF-β**	0.6297	0.7980	0.7044	3	0.7107	0.0487
**siNox4#2**	0.0900	0.0323	0.0437	3	0.0553	0.0176
**siNox4#2 + TGF-β**	0.4361	0.4836	0.5514	3	0.4904	0.0335

(D) Data underlying Fig 6B. Ratio Palladin 90 kDa/Tubulin.

Samples	Exp 1	Exp 2	Exp 3	n	m	SEM
**siNeg**	0.7295	0.5989	0.7240	3	0.6841	0.0427
**siNeg + TGF-β**	1.0107	1.0756	1.2552	3	1.1139	0.0731
**siNox4B#1**	0.5411	0.3731	0.3061	3	0.4067	0.0699
**siNox4B#1 + TGF-β**	0.7897	0.8505	0.8732	3	0.8378	0.0249
**siNox4#2**	0.5071	0.5076	0.4268	3	0.4805	0.0269
**siNox4#2 + TGF-β**	0.6725	0.8450	0.6653	3	0.7276	0.0587

F7 Dataset, Data underlying Fig 7.

(A) Data underlying Fig 7A. Molecular weight change of MRTF-A.

Samples	Exp 1	Exp 2	Exp 3	n	m	SEM
**siNeg**	0.7600	0.7600	0.7600	3	0.7600	0.0000
**siNeg + TGF-β**	8.8391	16.3770	7.2095	3	10.8085	2.8237
**siPalladin**	2.7925	1.0116	0.5504	3	1.4515	0.6836
**siPalladin + TGF-β**	3.9347	1.5160	3.0910	3	2.8472	0.7088

(B) Data underlying Fig 7B. Ratio SMA/Tubulin.

Samples	Exp 1	Exp 2	Exp 3	Exp 4	n	m	SEM
**siNeg**	0.4301	0.3001	0.4552	0.2345	4	0.3550	0.0526
**siNeg + TGF-β**	1.1661	0.7434	1.0491	1.3150	4	1.0684	0.1212
**siMRTF**	0.3154	0.6225	0.3327	0.3108	4	0.3954	0.0759
**siMRTF + TGF-β**	0.3506	0.5962	0.4251	0.4020	4	0.4435	0.0532

(C) Data underlying Fig 7C. Ratio CNN/Tubulin.

Samples	Exp 1	Exp 2	Exp 3	n	m	SEM
**siNeg**	0.2818	0.1201	0.1362	3	0.1794	0.0514
**siNeg + TGF-β**	0.7373	1.0711	0.9804	3	0.9296	0.0996
**siMRTF**	0.2457	0.0997	0.1444	3	0.1633	0.0432
**siMRTF + TGF-β**	0.5466	0.5205	0.5505	3	0.5392	0.0094

(D) Data underlying Fig 7D. Ratio Palladin 140 kDa/Tubulin.

Samples	Exp 1	Exp 2	Exp 3	n	m	SEM
**siNeg**	0.0457	0.1239	0.2055	3	0.1250	0.0461
**siNeg + TGF-β**	0.6864	0.4274	0.4344	3	0.5161	0.0852
**siPalladin**	0.0459	0.1239	0.0261	3	0.0653	0.0298
**siPalladin + TGF-β**	0.0456	0.1484	0.1577	3	0.1172	0.0359

(E) Data underlying Fig 7D. Ratio Palladin 90 kDa/Tubulin.

Samples	Exp 1	Exp 2	Exp 3	n	m	SEM
**siNeg**	0.8498	0.9431	0.8407	3	0.8779	0.0327
**siNeg + TGF-β**	1.2489	1.3065	1.1810	3	1.2455	0.0363
**siPalladin**	0.2275	0.1645	0.2119	3	0.2013	0.0190
**siPalladin + TGF-β**	0.2428	0.1550	0.3354	3	0.2444	0.0521

(F) Data underlying Fig 7D. Ratio SMA/Tubulin.

Samples	Exp 1	Exp 2	Exp 3	n	m	SEM
**siNeg**	0.1088	0.3806	0.1804	3	0.2233	0.0814
**siNeg + TGF-β**	1.0440	0.6434	0.8591	3	0.8488	0.1158
**siPalladin**	0.1429	0.2316	0.2194	3	0.1980	0.0278
**siPalladin + TGF-β**	0.3216	0.3616	0.3584	3	0.3472	0.0129

(G) Data underlying Fig 7E. Ratio CNN/Tubulin.

Samples	Exp 1	Exp 2	Exp 3	n	m	SEM
**siNeg**	0.2818	0.1201	0.1362	3	0.1794	0.0514
**siNeg + TGF-β**	0.7373	1.0711	0.9804	3	0.9296	0.0996
**siPalladin**	0.2457	0.0997	0.1444	3	0.1633	0.0432
**siPalladin + TGF-β**	0.5466	0.5205	0.5505	3	0.5392	0.0094
